# NK4 Regulates Laryngeal Squamous Cell Carcinoma Cell Properties and Inhibits Tumorigenicity by Modulating the DKK1/Wnt/β-Catenin Axis

**DOI:** 10.3389/fonc.2021.783575

**Published:** 2021-12-14

**Authors:** Shoukai Zhang, Hulai Wei, Xiaoqin Ha, Yueyu Zhang, Yufen Guo

**Affiliations:** ^1^ The Second School of Clinical Medicine, Lanzhou University, Lanzhou, China; ^2^ Otolaryngology-Head Neck Surgery, Gansu Provincial People’s Hospital, Lanzhou, China; ^3^ School of Basic Medical Sciences, Lanzhou University, Lanzhou, China; ^4^ Laboratory, People’s Liberation Army Joint Logistics Support Force 940th Hospital, Lanzhou, China; ^5^ Otolaryngology-Head Neck Surgery, Lanzhou University Second Hospital, Lanzhou, China

**Keywords:** NK4, laryngeal carcinoma, proliferation, migration, apoptosis, tumorigenicity

## Abstract

**Objective:**

To investigate the effects of NK4 gene on the properties and tumorigenicity in laryngeal squamous cell carcinoma cell.

**Methods:**

Here, we used the attenuated Salmonella carrying the NK4 gene to transfect the AMC-HN-8 cells and detected the expression of NK4 by the real-time quantitative polymerase chain reaction (q RT-PCR). The properties of NK4 gene was determined by MTT method, cell scratch test, and flow cytometry. A nude mouse tumorigenesis model was used to evaluate the effect of NK4 gene on the growth of AMC-HN-8 cells *in vivo*. While a western blot assay was used to assess the expression of DKK1, Wnt1 and β-Catenin in nude mouse tumors.

**Results:**

qRT-PCR showed that the expression of NK4 in the transfection group was significantly higher than that in the control group (P<0.01), and the expression increased with the time of transfection. MTT results showed NK4 overexpression inhibited the proliferation of AMC-HN-8 cells, and the inhibitory activity no longer increased with increasing dose when 30% expression supernatant was added (P<0.01). Scratch experiment showed that NK4 overexpression decreased the cell migration ability (P<0.01). Annexin V/PI double staining experiment showed that NK4 gene induced AMC-HN-8 cell apoptosis (P<0.01), and cell cycle arrest in S phase (P<0.01). NK4 overexpression inhibited tumor formation ability of AMC-HN-8 cells *in vivo* (P <0.05). WB detection showed that the expression of DKK1 increased, Wnt1 and β-Catenin protein decreased after the high expression of NK4.

**Conclusions:**

NK4 gene inhibit cell proliferation and migration, while promote cell apoptosis, and induce cell cycle arrest in S phase of laryngeal carcinoma AMC-HN-8 cells. NK4 overexpression inhibit the tumorigenesis ability of AMC-HN-8 cells, which may be related to the regulation of DKK1/Wnt1/β-Catenin signal axis.

## Introduction

Laryngeal cancer is the most common malignancy in the head and neck, accounting for 1-5% of systemic malignancies, of which 96-98% are laryngeal squamous cell carcinoma (LSCC). A study in 2018 (1) estimated that 177,422 new cases of laryngeal cancer and 94,771 deaths. Surgery is still the most effective treatment. Adjuvant radiotherapy, chemotherapy and novel gene-targeted therapy have led to significant progress in the treatment of laryngeal cancer. In recent years, gene-targeted therapy has shown good development and application prospects in cancer treatment. Therefore, seeking new approaches to treat laryngeal cancer at the gene level has become a new challenge faced by otolaryngologists.

Hepatocyte growth factor (HGF) is a cytokine produced by many interstitial cells, such as fibroblasts and macrophages, and acts on the receptor tyrosine-protein kinase Met (c-Met) on the surface of tumor cells. As a splice variant of HGF, NK4 has a heavy chain consisting of an n-terminal domain and 4 kringle domains, which compete with HGF for the binding of c-Met, is a specific antagonist of HGF. NK4 inhibits not only the signal transduction of the HGF/c-Met system but also the formation, invasion and metastasis of tumors, and it also inhibits tumor angiogenesis independent of the HGF/c-Met pathway and promotes tumor cell apoptosis. Dickkopf-1 (DKK1) is a secreted protein of the DKK protein family and an inhibitor of the extracellular Wnt signal transduction pathway (2). The Wnt signal transduction pathway is related to the occurrence, development, metastasis and prognosis of a variety of tumors. The DKK protein family has a similar conserved cysteine domain, which inhibits the Wnt/β-catenin pathway by causing the degradation of proteasome β-catenin, inducing apoptosis and inhibiting cell proliferation (3). Research (4) showed that DKK1 gene is highly expressed in head and neck squamous cell carcinoma, and its high expression may be related to HPV status, age, pathological grade, and clinical stage.

Based on the dual antitumor properties of the NK4 gene and the high expression of Wnt1/β-Catenin signaling pathway in the head and neck, this study aims to explore the following issues: (1) NK4 expression in transfected LSCC AMC-HN-8 cells; (2) the effects of the NK4 gene on the proliferation, migration, apoptosis and cell cycle of LSCC AMC-HN-8 cells;(3) The effect of NK4 gene on the tumorigenesis ability of nude mice of laryngeal cancer cells, and to explore whether its DKK1/Wnt1/β-Catenin signaling pathway is involved in the effect of NK4 gene on laryngeal cancer. The exploration of these issues can provide a deeper understanding of the important role of the NK4 gene in the development and progression of LSCC and provide theoretical support for gene therapy for LSCC.

## 1 Materials and Methods

### 1.1 Cells, Strains, and Main Reagents

The attenuated Salmonella strain carrying the NK4 gene was kindly provided by Professor Xiaoqin Ha from the 940 Hospital of Lanzhou People’s Liberation Army Joint Logistics Support Force; the LSCC AMC-HN-8 cells were purchased from Beiner Biological Cell Bank; high-glucose Dulbecco’s modified Eagle medium (H-DMEM) was purchased from HyClone Inc.; fetal bovine serum (FBS) was purchased from Hangzhou Sijiqing Bioengineering Materials Co., Ltd.; 0.25% trypsin-ethylenediaminetetraacetic acid (EDTA) was purchased from Gibco; penicillin and streptomycin were purchased from Biyuntian Biotechnology Co., Ltd.; methyl thiazolyl tetrazolium (MTT) was purchased from Beijing Solarbio Biotechnology Company; dimethyl sulfoxide (DMSO) was purchased from Wuhan BOSTER Biological Technology Co., Ltd.; total RNA extraction reagent, RNA reverse transcription kits, and real-time quantitative polymerase chain reaction (qRT-PCR) reagents were purchased from Takara Co., Ltd.; PCR primers were synthesized by Takara Biotechnology Co.; and annexin V fluorescein isothiocyanate (FITC)/propidium iodide (PI) double staining reagent kits and apoptosis kits were purchased from Invitrogen, USA. 4-6 weeks old, weighing 16-18g BALB/c nude mice were purchased from Beijing Weitong Lihua Laboratory Animal Technology Co., Ltd., license number SCXK (Beijing) 2016-0006; rabbit anti-human Wnt1 monoclonal antibody, rabbit anti-human β-Catenin and mouse anti-human DKK1 monoclonal antibody were purchased from Abcam, rabbit anti-human NK4 monoclonal antibody were purchased from Affinity.

### 1.2 Preparation of Attenuated Salmonella Carrying NK4 Gene

#### 1.2.1 Acquisition of NK4 Gene

According to literature search, design NK4 mRNA sequence, design specific primers for amplification of NK4 cDNA (**Table 1**), extract total RNA from human placental tissue, reverse transcription into cDNA, amplify NK4 gene, and amplify NK4 gene by PCR The parameters are: 94°C 30 s, 54°C 30 s, 72°C 2 min, cycle 30 times; The amplified product was detected by 1.0% agarose gel electrophoresis, and the product size was 1460 bp.

**Table 1:** Primer list.

Gene name	Sequences 5’-3’	Size (bp)	Enzyme site
NK4	Forward: CTG GTCGAC ATG TGG GTG ACC AAA CTC Reverse: GCA GCGGCCGC TCAG ACT ATT GTA GGT GTG GT	1460	SalI NotI

#### 1.2.2 Construction of the Eukaryotic Co-Expression Vector Carrying the NK4 Gene

After the PCR product of NK4 was recovered from the gel, it was digested with SalI and Not I. At the same time, the PIRES-SEQ plasmid was also digested with SalI and NotI; after 3 hours of digestion, it was digested with 1% Agarose gel electrophoresis respectively recovered NK4 gene fragment (about 1.5kb) and linearized PIRES-SEQ plasmid fragment (about 6.1kb); the recovered NK4 gene fragment and linearized pIRES-SEQ plasmid had a molar ratio of 5~ Mix at 10:1, add T4 ligase and connection Buffer, ligate at 4°C for 12 h; transform DH5a competent cells, spread on LB agar plates containing X-Gal IPTG Amp for culture.

#### 1.2.3 Preparation of Attenuated Salmonella Ty21a Competent Cells

Attenuated Salmonella Ty21a (previously constructed by Professor Ha Xiaoqin of the 940th Hospital of the Joint Service Support Force of the Chinese People’s Liberation Army) inoculate the attenuated Salmonella Ty21a strain in 50mL LB liquid medium, shake culture to A525nm of about 0.6, collect the bacteria by centrifugation at 4°C 4000 rpm/min, and use pre-chilled sterile Wash the bacteria twice with deionized water and suspend in 1 mL of ice-cold sterile deionized water.

#### 1.2.4 Electrotransformation Process

Add 0.2 µg of plasmid pCMV-NK4-IRES to 200 µL of the above bacterial solution for electrotransformation, add 1 mL of SOC medium after electric shock, shake at 37°C for 45 minutes, and spread on an ampicillin-resistant LB plate. Incubate overnight at 37°C in an incubator.

#### 1.2.5 Screening and Identification of Attenuated Salmonella Carrying NK4 Gene

Take out the LB plate the next day, pick 10 monoclonal colonies from the culture plate, and inoculate them in 3 mL of LB medium containing ampicillin resistance (selection positive only), 37 Incubate with shaking at °C overnight (not more than 16 h). Take 2 mL of the bacterial solution and mix with glycerol the next day, then freeze it (containing 15% glycerol). The remaining 1 mL of the bacterial solution was centrifuged to collect the bacteria, and the plasmid was extracted with a small plasmid extraction kit. The extracted plasmid was detected by 1% agarose electrophoresis and then identified by PCR and Sal 1/NotIrestriction enzyme digestion.

### 1.3 Culture of LSCC AMC-HN-8 Cells

The cells were cultured in DMEM containing 10% FBS, 1% penicillin and streptomycin (100 U/mL penicillin and 100 μg/mL streptomycin) in a 37°C, 5% CO_2_ incubator. Cells were digested with 0.25% trypsin and passaged once every 3 d. The culture medium was changed once every 2 d. Cells in logarithmic growth phase were used for subsequent experiments.

### 1.4 Transfection of LSCC AMC-HN-8 Cells

LSCC AMC-HN-8 cells were cultured in H-DMEM containing 10% FBS. After the cell confluence reached 90% or higher, the cells were digested with 0.25% trypsin and passaged. One day before transfection, the cells were inoculated on 6-well culture plates at 6×10^5^ cells/well and were divided into a control group and NK4 group, with 3 wells for each group. The next day, attenuated Salmonella stains carrying NK4 were inoculated into 3 mL of Luria-Bertani (LB) broth. Cells were shaken at 37°C for 4 h. When the absorbance at 525 nm (A_525nm_) was approximately 0.6, the bacteria were collected by centrifugation (4000 rpm/min) at 4°C. The bacteria were washed twice with precooled sterile deionized (DI) water and then suspended in sterile DI water at the bacterial concentration of 1 × 10^8^/ml. Prior to transfection, cells in each group were washed with antibiotic-free DMEM twice. The prepared cells and bacteria were added into the culture wells of the transfection group at a ratio of 1:50 and incubated at 37°C for 1 h; then, the cells were washed with PBS twice and incubated in serum-free medium (containing 50 mg/L gentamicin) for 1 h, and last, complete medium with gentamicin (10 mg/L) was added for further culture.

### 1.5 NK4 Expression Detected by RT-PCR

At 24 h and 48 h after transfection, the LSCC AMC-HN-8 cells were harvested, and total RNA was extracted using Trizol according to the manufacturer’s instructions. Reverse transcription of RNA was performed according to the manufacturer’s instructions (Takara) to obtain complementary DNA (cDNA). Primer sequences for the NK4 gene (**Table 2**). The reverse transcription conditions were as follows: 42°C for 2 min, 37°C for 15 min, and 85°C for 5 s. The reverse transcription products were used as templates for PCR amplification; β-Actin was used as the internal reference. The PCR amplification conditions were as follows: step 1, 95°C for 30 s; step 2, 95°C for 5 s, 57°C for 35 s, and 72°C for 60 s, for a total of 45 cycles; and step 3, extension at 65°C for 5 min. The absorbance was read at each extension stage. Multiple wells were setup for each sample, and the average value was used for statistical analysis. Under the condition that the amplification efficiency was consistent, the 2^-ΔΔCT^ method was used to obtain the relative quantitative results. Agarose gel electrophoresis was performed on PCR products to detect the expected NK4 gene fragment (163 bp). The expression of the target gene was detected by performing agarose gel electrophoresis with the PCR products.

**Table 2:** Primer sequences for the NK4 gene.

Primer name	Primer sequence 5′-3′	Product length
NK4	GTGAATACTGCAGACCAATGTGCTA	163 bp
	GGTCAAATTCATGGCCAAATTC	
β-Actin	TGGCACCCAGCACAATGAA	186 bp
	CTAAGTCATAGTCCGCCTAGAAGCA	

### 1.6 Determination of Cell Proliferation Using the MTT Assay

The supernatant of laryngeal cancer AMC-HN-8 cells cultured 48h after transfection was collected according to the above 1.4 method, and the protein was extracted to identify the expression of NK4 protein. After the expression of NK4 protein was confirmed by Western blot, MTT was used to detect cell proliferation capacity on this basis: AMC-HN-8 cells in the logarithmic growth phase were inoculated into 96-well cell culture plates at 5 ×10^3^ cells/well and divided into a control group and an NK4 group. After a 12-h culture, the original culture medium was aspirated and discarded. Serum-free DMEM (100 μl/well) and the cell culture supernatant after 48 h of transfection were added into the wells of the NK4 group, with percentages of expression supernatant accounting for 5%, 10%, 15%, 20%, 25%, 30%, and 35% of the total culture volume. The control group had 3 wells, and there were also 3 wells for each expression supernatant percentage in the NK4 group. In the control group, only serum-free DMEM (100 μl/well) was added. After incubating at 37°C for 48 h, 10 μl of MTT (5 mg/ml, prepared with PBS and then after filtered and sterilized) solution was added to each well, followed by incubation in a cell incubator for 4 h. Then, 100 μl of DMSO was added, and the cells were shaken for 15 min. The optical density (OD) at 570 nm was measured. A cell growth curve was plotted with OD as the vertical axis, and the inhibition rate of cell growth was calculated. Y = 1-A/B × 100%, where Y is the cell inhibition rate, A is the OD of the experimental group, and B is the OD of the control group. The experiment was repeated 3 times.

### 1.7 Cell Migration Measured by the Scratch Assay

Cells in logarithmic growth phase were inoculated into 6-well plates and divided into a control group and an NK4 group. After the cells reached more than 90% confluence, a 10-μL sterile tip was used to scratch the monolayer of the cells at the bottom of each well before transfection. The cells were transfected according to the method described in *Section 1.3.* At 0, 24, 48, and 72 h after transfection, healing was observed under a microscope and photographed. ImageJ software was used to measure the scratch area and calculate cell migration ability, as follows: migration ability = (scratch area at 0 h - scratch area at 24 h/48 h/72 h)/scratch area at 0 h × 100%. The experiment was repeated 3 times.

### 1.8 Cell Cycle Measured by Flow Cytometry

Cells in logarithmic growth phase were inoculated into 6-well plates at 6×10^5^ cells/well and were divided into a control group and an NK4 group. The cells were transfected according to the method described in *Section 1.3.* A cell suspension was prepared after 48 h of culture. Cells were fixed in 75% ethanol overnight, washed with PBS once, and stained with PI. After incubation at 4°C in the dark for 30 min, flow cytometry was performed, and the percentage of cells in each cell cycle phase was calculated using Modifit software. The experiment was repeated 3 times.

### 1.9 Cell Apoptosis Measured by Flow Cytometry

Cells in logarithmic growth phase were inoculated into 6-well plates at 6×10^5^ cells/well and were divided into a control group and an NK4 group. The cells were transfected according to the method described in *Section 1.3.* After 24 h, 48 h, and 72 h of culture, the cells were digested with trypsin and centrifuged, and the supernatant was discarded. Two hundred microliters of 1× binding buffer was added, and the cells were resuspended, followed by the addition of 5 μL of annexin V-FITC and 5 μL of PI. The reaction was carried out at room temperature for 15 min in the dark. Apoptosis was detected by flow cytometry. The experiment was repeated 3 times.

### 1.10 Establishment of Subcutaneous Laryngeal Carcinoma Xenograft Model in Nude Mice

Take 30 nude mice, inoculate the logarithmic growth phase of laryngeal cancer cells at a cell density of 1 × 10^7^/ml on the skin of the right side of the axilla of the nude mice, and inoculate at a volume of 0.2 mL for each nude mouse. When a tumor nodule is visible to the naked eye, measure the longest diameter of the tumor (a) and the short diameter (b) perpendicular to it with a vernier caliper every 3 days. The final tumor volume is calculated according to the formula V = 1/2ab^2^, when the tumor volume Reached about 100 mm3, they were randomly divided into a control group and a treatment group, with 15 animals in each group. Suspend the attenuated Salmonella carrying the NK4 gene in 10% NaHCO3, adjust the cell concentration to 1×10^9^ cells/ml, and take the corresponding bacteria 0.1 ml with gastric tube feeding once a week for 4 times in total, the control group Take an equal volume of 0.1M PBS buffer solution. One week after the treatment, the nude mice were sacrificed by cervical vertebrae removal method, the subcutaneous tumor nodules were stripped, and the tumor weight was weighed after taking pictures. The tumor tissue was fixed with 10% neutral formalin for routine HE and immunohistochemical staining.

### 1.11 Western Blot Detection of DKK1, Wnt1, β-Catenin Protein

RIPA lyses the tumor to extract the protein. After the protein concentration is determined by the BCA method, the protein loading buffer is added and the protein is boiled in boiling water for 10 minutes to denature the protein. Equipped with 10% separation gel and 5% concentrated gel, the concentrated gel runs at a constant pressure of 80 V for 30 minutes, and the separation gel runs at a constant pressure of 110 V for 90 minutes. Cut out the glue of the corresponding molecular weight and transfer it for 30 to 90 min at a constant current of 200mA at 4°C. 5% milk blocking solution was sealed at room temperature for 1 h. Incubate the primary antibody overnight at 4°C. After washing the excess primary antibody with TBST the next day, incubate the secondary antibody at room temperature for 1 hour, add ECL luminescent reagent dropwise, and expose and image in the imager. Image J software analyzes the gray value of each band. The experiment was repeated 3 times.

### 1.12 Statistical Methods

All data were processed first using Excel, and statistical analysis was performed using SPSS 23.0 software. The data are expressed as the mean ± standard deviation (*x̄* +*S*). The paired sample t test was used to compare the means between 2 groups. One-way analysis of variance (ANOVA) was used for comparisons among multiple groups. P<0.05 was considered statistically significant.

## 2 Results

### 2.1 Morphological Changes in LSCC AMC-HN-8 Cells After Transfection

**Figure 1:**
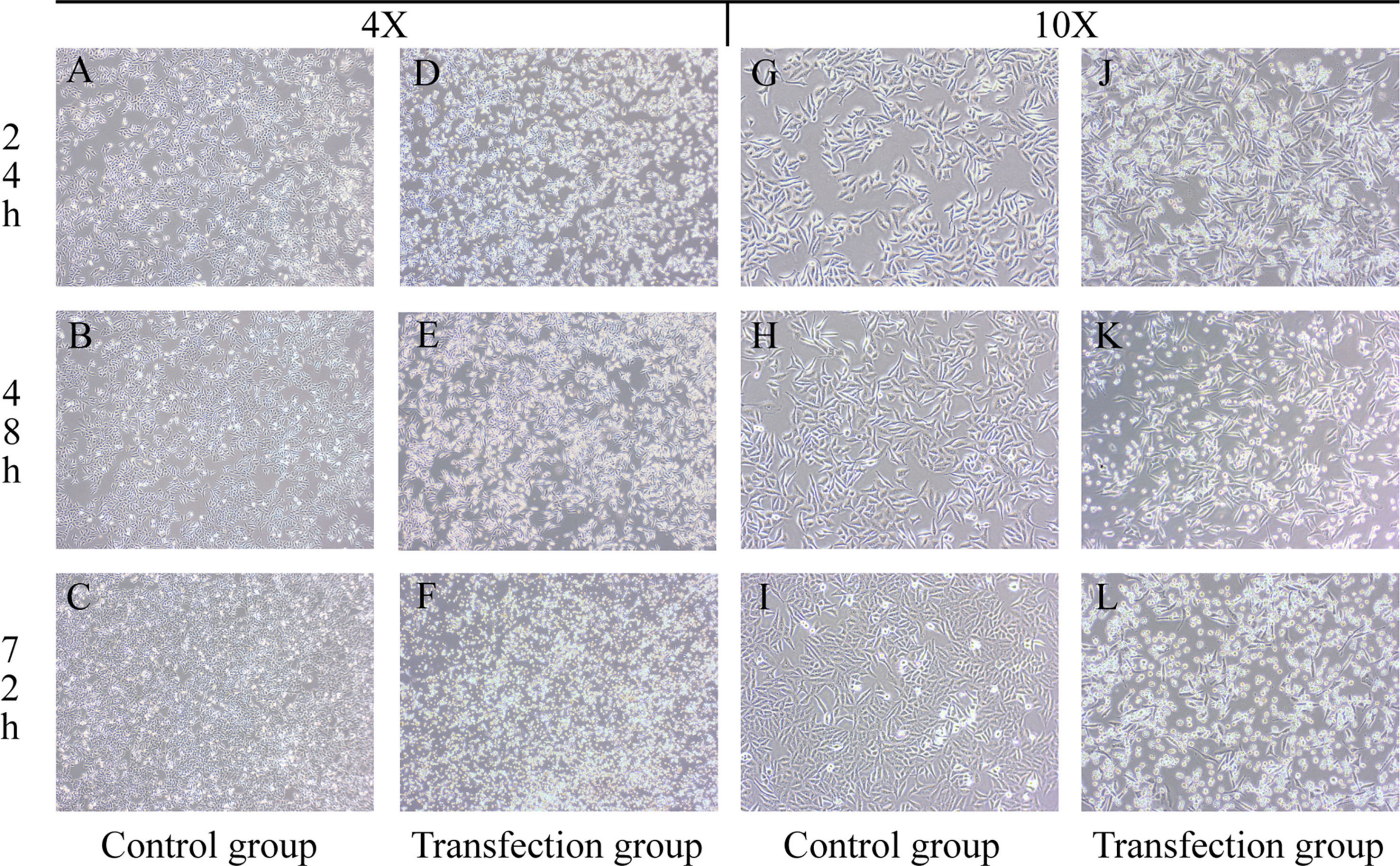
Groups **(A–C, G–I)** are the control groups; Groups **(D–F, J–L)** are the transfection groups.

The growth state of cells was directly observed under a microscope. After transfection, the normal morphology of LSCC AMC-HN-8 cells was destroyed, and the proliferation and growth were inhibited. Microscopic observation showed that as the transfection time increased, apoptotic cells increased, and the cell morphology changed significantly. As shown in **Figure 1**, after the cells in the control group were cultured for 24 h, 48 h, and 72 h, the number of cells increased, adherent growth was vigorous, and the adjacent cells grew and fused together, with a full cytoplasm, clear cell profile, spindle or polygon shape, and well dispersed. After the transfected LSCC AMC-HN-8 cells were cultured for 24 h, 48 h, and 72 h, the number of visible cells gradually decreased, the cell morphology gradually became cord-like, the connection between cells was relaxed, the cells became nonadherent, granular substances increased, and the number of apoptotic cells increased.

### 2.2 Expression of the NK4 Gene in LSCC AMC-HN-8 Cells

**Figure 2:**
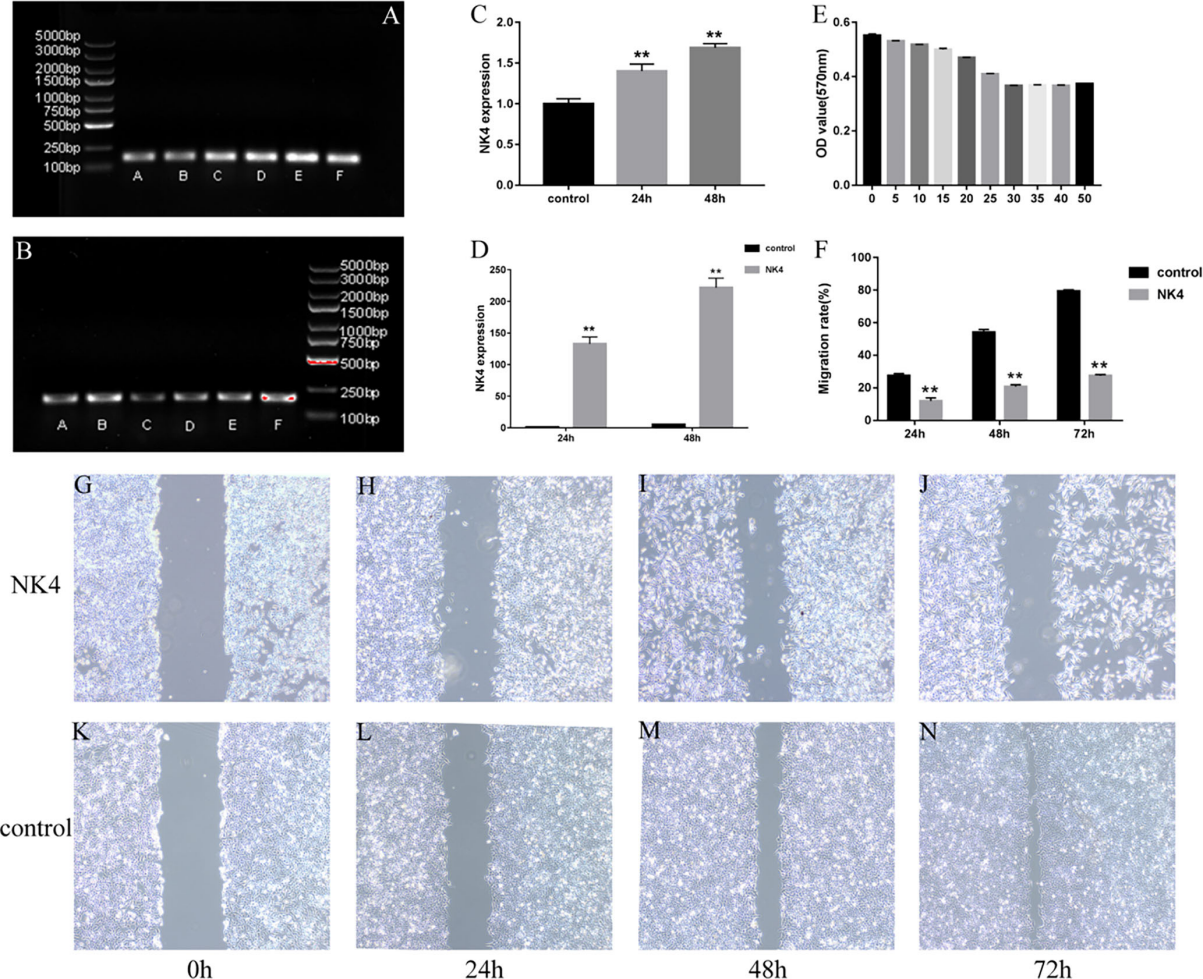
**(A)** NK4 gene fragment; **(B)** β-actin gene (the internal reference); **(C)** Grayscale value analysis of electrophoretic gel images (after PCR); **(D)** NK4 gene expression; **(E)** NK4 inhibits the proliferation of laryngeal cancer cells; **(F)** the wound healing of cells in the control group and NK4 group at 24h, 48h, and 72h; **(G–N)** the wound healing of cells in the control group and NK4 group at 24h, 48h, and 72h. **P < 0.01.

The amplified NK4 gene and the β-actin fragment (the internal reference) were subjected to agarose gel electrophoresis. In the gel system, the NK4 fragment band (163 bp) and the β-actin fragment band (186 bp) were identified. The size of the NK4 fragment after PCR amplification was consistent with the theoretical size of the NK4 fragment, and the band intensity increased with the transfection time. The results are shown in **Figures 2A–C** (P<0.01). The relative expression level of NK4 in each group of cells was calculated using the 2^- ΔΔCt^ method. The RT-PCR results showed that NK4 expression was significantly increased in the transfection group; the difference was statistically significant (P<0.01, **Figure 2D**).

### 2.3 Effect of NK4 Gene Inhibition on the Proliferation of LSCC AMC-HN-8 Cells

**Figure 3:**
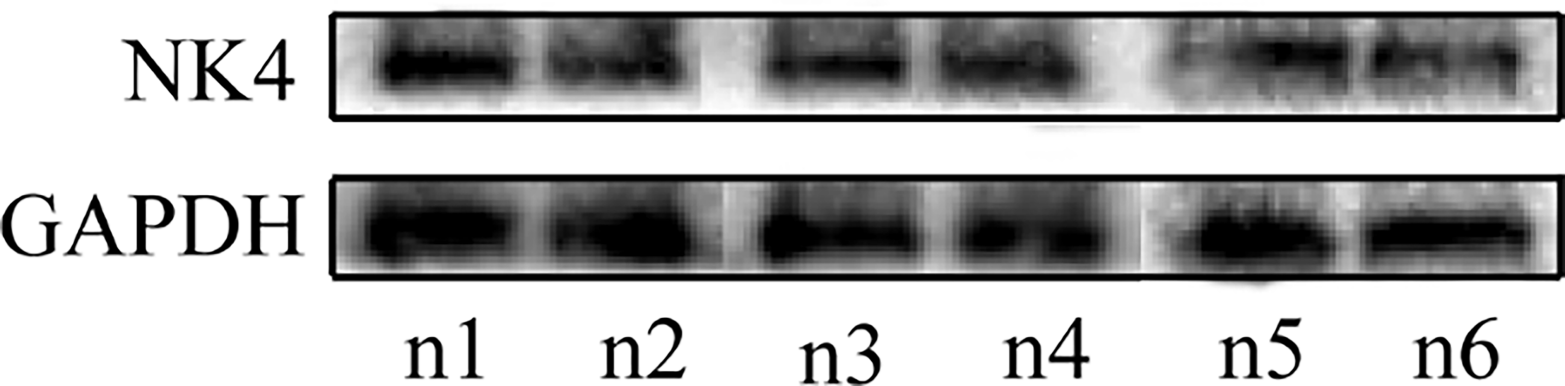
n1-n6: the expression of NK4 protein.

After the expression of NK4 (26kD)protein was confirmed by Western blot, The results are shown in **Figure 3**. The MTT assay results showed that the expression products of the transfected cells significantly inhibited the proliferation activity of AMC-HN-8 cells in a percentage-dependent manner. After the percentage of expression supernatant reached 30%, the inhibitory effect no longer increased as the percentage increased, and the inhibition rate was 33.56% (**Figure 2E**, P<0.01).

### 2.4 Effects of NK4 Gene Inhibition on the Migration Ability of LSCC AMC-HN-8 Cells

The scratch assay was used to determine the effect of NK4 gene inhibition on the migration ability of LSCC AMC-HN-8 cells (**Figures 2F, G–N**). The scratch wound healing rates of transfected AMC-HN-8 cells at 24 h, 48 h, and 72 h after scratching were 11.97% ± 4.40%, 20.75% ± 3.55%, and 27.73% ± 2.56%, respectively, lower than those in the control group (24.82% ± 3.01%, 54.15% ± 5.11%, and 79.37% ± 2.45%, respectively); the differences were statistically significant (P<0.01). The scratch assay results showed that NK4 inhibited the migration of AMC-HN-8 cells.

### 2.5 Effect of the NK4 Gene on LSCC AMC-HN-8 Cell Apoptosis

**Figure 4:**
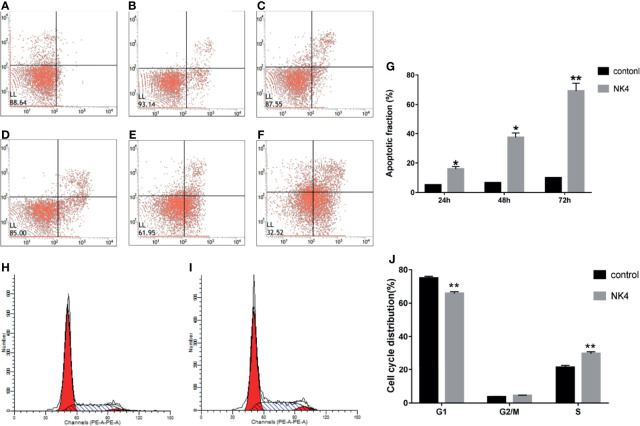
**(A)** 24h control group; **(B)** 48h control group; **(C)** 72h control group; **(D)** 24h NK4 group; **(E)** 48h NK4 group; **(F)** 72h NK4 group; **(G)** Apoptosis rate of NK4 group at 24h, 48h, 72h; **(H)** Percentage of cells in each cell cycle phase (control group); **(I)** Percentage of cells in each cell cycle phase (NK4 group); **(J)** Percentage of cells in each cell cycle phase *P < 0.05 **P < 0.01.

Apoptosis of the cells in each group was detected by flow cytometry after annexin V-FITC and PI double staining. The results are shown in **Figures 4A–G**. The cell apoptosis rates in the NK4 group at 24 h, 48 h, and 72 h were 15.99% ± 2.83%, 37.37% ± 5.29%, and 69.21% ± 9.13%, respectively, which were all higher than those in the control group; the differences were statistically significant. The NK4 gene can promote AMC-HN-8 cell apoptosis.

### 2.6 Effect of the NK4 Gene on the Cell Cycle of LSCC AMC-HN-8 Cells

The flow cytometry results (**Figures 4H–J**) showed that the proportion of cells in G_1_ phase in the NK4 group (65.78% ± 3.24%) was lower than that in the control group (74.98% ± 3.20%) and that the proportion of cells in S phase (29.77% ± 3.29%) was higher than that in the control group (21.41% ± 3.96%); the differences were statistically significant (P<0.01). The proportions of cells in G_2_/M phases were 4.43% ± 0.72% and 3.58% ± 1.01%, respectively; the differences were not statistically significant (P>0.05). These results indicated that the NK4 gene induce S phase arrest.

### 2.7 Tumor Volume Growth Curve of Transplanted Tumor in Nude Mice

**Figure 5:**
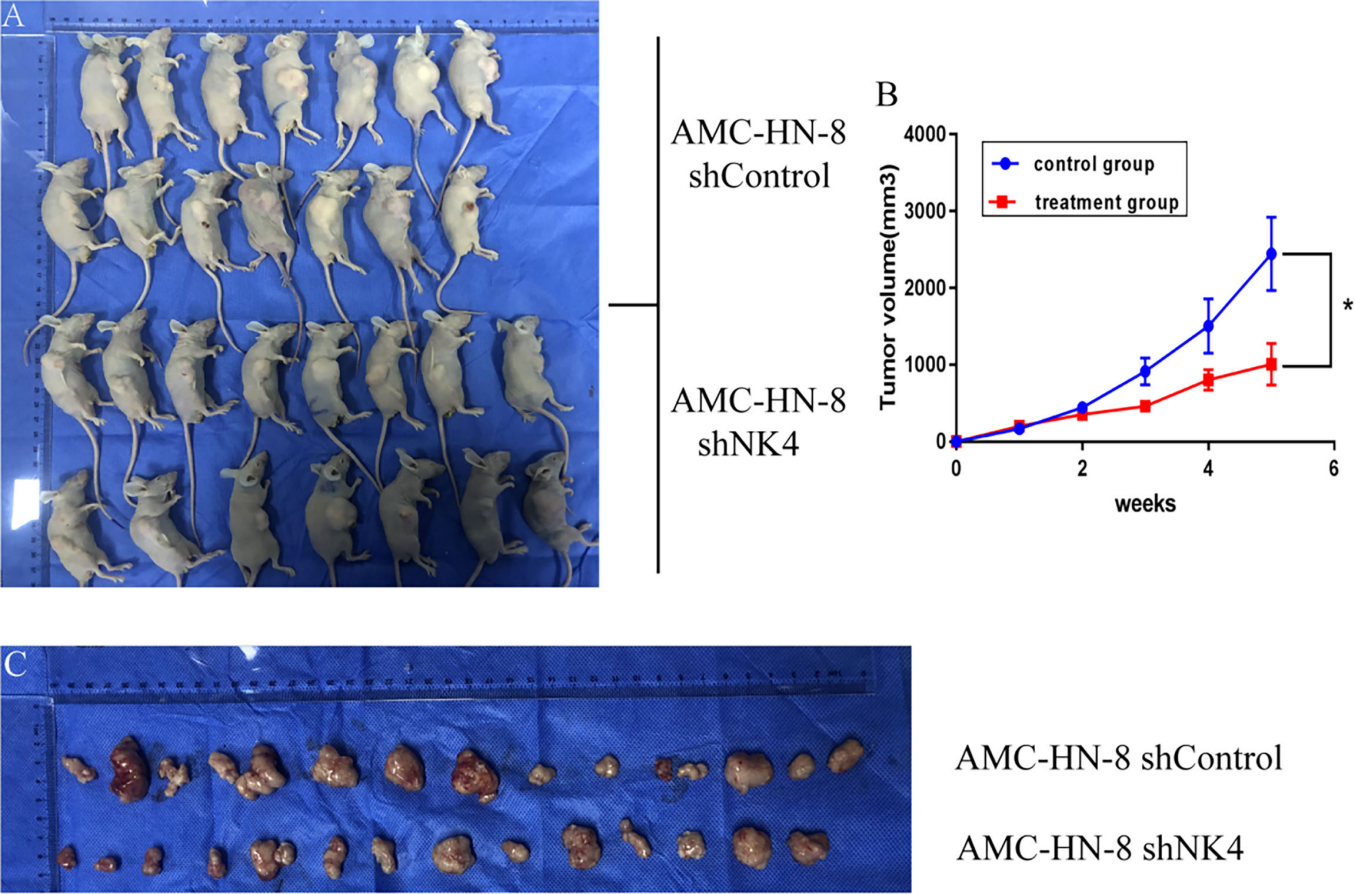
**(A)** Tumor formation in nude mice; **(B)** Growth curve of transplanted tumor; **(C)** Change in volume and size of transplanted tumor. *P < 0.05.

Subcutaneous nodules can be seen 5 days after inoculation. 1 animal in the control group died on the 20th day, and 14 mice remained; all 15 nude mice in the treatment group survived. The tumor volume was measured, recorded and calculated every 3 days, and the growth curve was drawn. The results showed that the subcutaneous tumor volume in the treatment group was smaller than that in the control group, and the difference between the two groups was significant (P<0.05), as shown in **Figure 5**.

### 2.8 Western Blot Method to Detect Protein Expression in Tumor Tissues of Nude Mice

**Figure 6:**
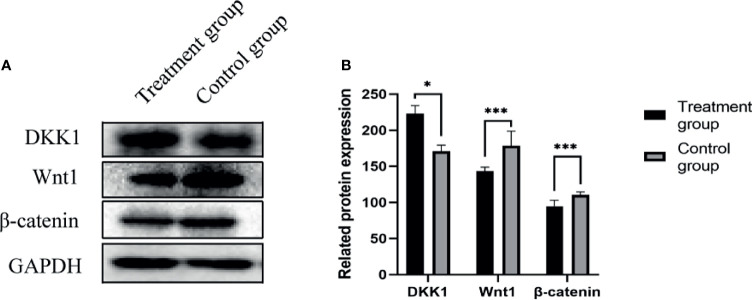
**(A)** DKK1, Wnt1, β-Catenin protein expression band; **(B)** DKK1, Wnt1, β-Catenin protein expression level. *P < 0.05, ***P < 0.001.

Western blot method was used to detect the expression of various proteins in the tumors of the nude mice in the negative control group and the treatment group. The expressions of DKK1(35kD) increased, Wnt1(41kD) and β-Catenin(92kD) protein in the tumor tissues of the treatment group were significantly reduced, as shown in **Figure 6**.

## 3 Discussion

At present, surgery is the major treatment strategy for laryngeal cancer. Most gene-targeted therapies for laryngeal cancer are at the experimental exploration stage. For patients with advanced laryngeal cancer, accompanying serious complications affect the prognosis; therefore, it is urgent to explore new auxiliary methods for treating laryngeal cancer. Gene-targeted therapy for tumors aims to limit the inhibitory effect of genes on specific target cells, tissues or organs, so as to increase gene sensitivity, improve curative effects and reduce side effects. The HGF/c-Met receptor pathway plays a role in tumor growth, metastasis, invasion, and angiogenesis (5). NK4 is a competitive antagonist isolated from HGF, consisting of an n-terminal domain and 4 kringle domains, and can counteract the association between HGF and c-Met. NK4 not only acts like an antagonist, inhibiting HGF/c-Met induced tumor growth, metastasis and invasion, but also inhibits vascular endothelial growth factor (VEGF) - and basic fibroblast growth factor (bFGF)-induced tumor angiogenesis, which is independent of the HGF/c-Met pathway, and ultimately causes the tumor cell apoptosis. NK4 gene therapy can inhibit growth, invasion, metastasis and angiogenesis in a variety of tumors, including breast cancer, prostate cancer, colon cancer, mesothelioma, lung cancer, pancreatic cancer and brain cancer (6). In experimental models of different cancers, NK4 gene therapy inhibited the activation of the Met receptor, which is related to the inhibition of tumor invasion and metastasis. Similarly, NK4 gene therapy inhibited tumor angiogenesis and thereby inhibited angiogenesis-dependent tumor growth. The NK4 gene deserves further research and attention as a potential cancer treatment target. Sawatsubashi et al. (7) used immunohistochemistry to study the pathological tissues of 82 cases of laryngeal cancer and found that HGF/c-Met was highly expressed in laryngeal squamous cell carcinoma, and the high expression of HGF/c-Met was positively correlated with the degree of lymph node metastasis. The research results of Haddad et al. (8) are consistent with their research results. Yucel et al. (9) found in a study of 60 cases of supraglottic carcinoma (including 30 cases of cervical lymph node metastasis) that 90% of tumors were High expression of c-Met; c-Met is highly expressed in 83% of metastatic lymph nodes. Choe J Y et al. (10) confirmed that the high expression of C-Met is related to the primary site of the tumor; the hypopharynx has the highest expression, followed by the oral cavity, pharynx and nasal cavity. Squamous cell carcinoma expresses c-Met more frequently than undifferentiated carcinoma. Jiang M et al. (11) used immunohistochemical methods to detect c-Met protein in 71 cases of primary laryngeal carcinoma. The expression rate of c-Met in glottic squamous cell carcinoma of laryngeal carcinoma was 69.0%. There were patients with high c-Met expression. The tendency of the tumor to recur. In summary, the above studies have confirmed that the expression of HGF/c-Met in both serology and histology is an important factor affecting the occurrence and development of laryngeal squamous cell carcinoma, and is related to the pathological staging, disease prognosis, and survival rate of patients with laryngeal carcinoma. There is a correlation in such aspects. It can be inferred from this that NK4 antagonizes HGF/c-Met may inhibit the proliferation of laryngeal cancer cells and the formation of lymph vessels and microvessels in laryngeal cancer tissues, and block the occurrence, development and lymph node metastasis of laryngeal cancer.

This study used an attenuated Salmonella strain carrying the NK4 gene to transfect the LSCC cell line AMC-HN-8 to further investigate the effect of NK4 on the biological functions of AMC-HN-8 cells *in vitro*. NK4 gene delivery was mediated by the attenuated Salmonella vector. The attenuated Salmonella strain carrying the NK4 gene was successfully transfected into the LSCC AMC-HN-8 cells. Clinical common gene vectors include viral vectors, bacterial vectors and some non-bacterial vectors. Cai et al. (12) used bone marrow mesenchymal stem cells (BMSCs) as a vector for the NK4 gene and confirmed that NK4 gene-modified BMSCs can inhibit the growth, metastasis, and angiogenesis of liver cancer cells. BMSCs have the ability to migrate to tumor sites and integrate into the tumor vascular wall, with low immunogenicity. Adenovirus-mediated NK4 gene therapy can inhibit the mesothelioma cancer stem-like cells (13). Adenoviral NK4 (Ad-NK4) can effectively inhibit the activity, invasiveness and tumorigenicity of human multiple myeloma (MM) cells (14). NK4 expression can significantly decrease the activity of Met and AKT and inhibit the activity, invasiveness, and tumorigenicity of MM cells. These results are consistent with the previously reported effects of NK4. Currently, attenuated Salmonella strains are the most widely studied. Attenuated Salmonella strains are safe, invasive and tumor-targeting vectors for antitumor gene delivery (15), and the use of attenuated Salmonella strains carrying interleukin (IL)-2 (16), Fas ligand (FasL), IL-18 (17) and fms-like tyrosine kinase 3 (FLT3) ligand (18) led to great progress in antitumor research. In recent years, the attenuated Salmonella TPIN carrying NK4/IL-2, prepared by Professor Xiaoqin Ha, was shown to efficiently transfect tumor cells *in vitro* to express the target proteins IL-2 and NK4, and the expression products inhibited HepG2 cell proliferation and migration and inhibited the formation of vessels in chick chorioallantoic membrane. It was already used for the treatment of liver cancer (19) and its inhibitory effect was confirmed.

This study showed that an attenuated Salmonella strain carrying NK4 was successfully transfected into LSCC AMC-HN-8 cells. With increasing transfection time, the number of cells decreased, and the morphology of cells changed, indicating that the NK4 gene can affect the proliferation of AMC-HN-8 cells. QRT-PCR was used to detect the expression of the NK4 gene in AMC-HN-8 cells 24 and 48 h after transfection. The results showed that NK4 was highly expressed in AMC-HN-8 cells. MTT assay results confirmed that the NK4 gene affected cell proliferation. Given that in 24 hours the cells round up, cells die at 48 hours the inhibition of cell migration could be an indirect result of the cell death. In fact, in our experiment in order to shun the disturbing to cell migration caused by inhibited or dead cells, in the scratch assay the equal number of viable cells were used in Control group and NK4 group, respectively, and the viable cells were counted by typan blue exclusion. MTT test results showed that the expression products of the transfected cells significantly inhibited the proliferation activity of AMC-HN-8 cells in a percentage dependent manner with statistical difference. The NK4 gene is considered to inhibit cell proliferation. Studies have shown (20) that NK4 is used in the treatment of prostate cancer, The mechanism by which NK4 promotes apoptosis in DU145 cells maybe its inhibitory effect on HGF-induced proliferation, leading to increased apoptosis. The antitumor effect of NK4 on DU145cells was specific for the HGF signal. Activation of Ras/ERK/MAPK (mitogen-activated protein kinase) changes the expression/activation of cell cycle regulators that affect cell proliferation. Activator that control cell migration and invasion, whereas PI3K/Akt activation by HGF mediates cell survival and resistance to apoptosis (21). NK4 gene is believed to inhibit the proliferation of laryngeal cancer cells. The possible mechanism is consistent with the above-mentioned studies, which promotes cell apoptosis and inhibits cell proliferation. The scratch assay results showed that at 72 h after transfection, the cells on either side of the scratch in the control group were close to each other, while the scratch in the transfection group was still wide, suggesting that the NK4 gene had an inhibitory effect on the migration of AMC-HN-8 cells. The inhibition of cell proliferation is known to be associated with the induction of apoptosis and cell cycle arrest. In this study, flow cytometry was used, and the results showed that the NK4 gene affected apoptosis and induced S phase arrest in AMC-HN-8 cells at 48 h after transfection. We found that NK4 inhibited the proliferation of AMC-HN-8 cells and induced apoptosis through S phase arrest. This is inconsistent with the results of previous studies showing that NK4 induces growth inhibition in other types of cells by causing G_1_ phase arrest (14, 22). Preliminary *in vivo* experimental studies confirmed the effect of NK4 gene on the proliferation, migration and apoptosis of laryngeal cancer cells, and further *in vivo* experiments to explore its mechanism. We established a nude mouse model of laryngeal cancer and observed the tumor volume changes in nude mice after gavage treatment with attenuated Salmonella carrying the NK4 gene. The results showed that after treatment, the tumor volume in the treatment group was smaller than that in the control group. In this study, we used oral gavage to investigate whether Salmonella strains containing the NK4 gene can inhibit the growth of laryngeal cancer cells in nude mice. The main reason is that pre-tumor gene vaccine is the most effective biological treatment method at this stage. Attenuated Salmonella has the unique advantage of delivering therapeutic genes in tumor treatment and has become a hot spot for tumor gene vaccine research. The virulence gene mutations of Salmonella are induced through physical, chemical, genetic engineering and other techniques to obtain attenuated Salmonella that maintain invasiveness. Because its preparation method is mature, easy to expand, and stable in nature, oral administration does not affect the therapeutic effect, and it can be inoculated into tumor patients and animals on a large scale. Attenuated Salmonella mediated the target gene expression *in vivo* with high efficiency; specific aggregation in target tumor tissues and cells shows targeting; it can stimulate the body to quickly respond to the corresponding cells and humoral. Attenuated Salmonella can be used as a therapeutic gene carrier and its expression in tissues is highly targeted. Since this bacterium itself is an intestinal parasite, the amount of expression in the digestive tract can meet the needs of treatment. The experimental conclusion is consistent with the study of Theys, Sznol et al (19, 23, 24). By Western blot Method to detect the expression of DKK1 increased, Wnt1 and β-Catenin protein in tumor tissues are reduced, and previous studies have confirmed that the Wnt1/β-catenin signaling pathway is abnormally activated in a variety of human malignant tumors, and promotes tumor invasion and metastasis. The Wnt/β-catenin signaling pathway is involved in various key cell functions, such as stem cell regeneration and organogenesis (25). In various types of malignant tumors, including breast, lung, and hematopoietic system, Wnt activation has been found, and it has been proven to help tumor recurrence (26). When the Wnt ligand binds to the Frizzled (Frizzled, Fz)/low density lipoprotein-related receptor protein (LRP5/6) complex, the activity of destroying the complex is inhibited, and β-catenin in the cytoplasm is stabilized and continuously Accumulate, translocate to the nucleus after reaching a certain concentration and combine with the transcription factor T cytokine factor 4 (TCF4)/lymphatic enhancer factor 1 (LEF1) to recruit different nuclear regulatory factors, thereby activating the expression of related genes downstream of the Wnt pathway to generate various types of Inflammatory mediators and fibrotic factors induce disease (27, 28). The activation of this pathway is related to a variety of chronic kidney disease, cancer, osteoporosis, degenerative diseases, etc. (29). It has also been confirmed in head and neck tumors that Wnt/β-catenin signaling can accelerate the tumor progression of nasopharyngeal carcinoma and esophageal squamous cell carcinoma (30). Therefore, inhibition of Wnt/β-catenin signaling pathway may be used as a kind of laryngeal cancer. In addition, DKK1 gene can be activated by competitively binding to LRP5/6 receptors to negatively regulate Wnt protein-mediated signal transduction. This experiment confirms that NK4 gene the anti-tumor effect may be related to the regulation of DKK1/Wnt/β-Catenin related pathways, but further experiments are needed to prove this conclusion.

In summary, NK4 can inhibit the proliferation and migration of AMC-HN-8 cells, induce the cell cycle S phase arrest, and has a significant impact on cell apoptosis. *In vivo* experiments have also confirmed that NK4 inhibits tumor formation in nude mice, and it may be related to the regulation of DKK1/Wnt1/β-Catenin related pathways. Therefore, the NK4 gene can be used as a new target for the treatment of laryngeal cancer. The limitation of this study is that the mechanism that the NK4 gene affects the occurrence and development of laryngeal cancer may be related to the regulation of the DKK1/Wnt1/β-Catenin related pathways, which needs to be demonstrated by further experiments. In follow-up experiments, we will further explore how the NK4 gene plays a specific role in laryngeal cancer, laying a solid theoretical foundation for the research and preparation of effective anti-tumor biologic drugs.

## Data Availability Statement

The original contributions presented in the study are included in the article/supplementary materials. Further inquiries can be directed to the corresponding author.

## Ethics Statement

The animal study was reviewed and approved by the Ethics Committee of Gansu Provincial People’s Hospital.

## Author Contributions

SZ is responsible for the conduct of the experiment and the writing of the paper, HW is responsible for guiding the conduct of the experiment and the writing of the thesis, XH is responsible for guiding the conduct of the experiment and the writing of the thesis, YZ is responsible for assisting in the conduct of the experiment and the writing of the paper, YG is responsible for guiding the conduct of the experiment and the writing of the thesis. All authors contributed to the article and approved the submitted version.

## Funding

Science and Technology Plan Project of Gansu Province 20JRIORA367.

## Conflict of Interest

The authors declare that the research was conducted in the absence of any commercial or financial relationships that could be construed as a potential conflict of interest.

## Publisher’s Note

All claims expressed in this article are solely those of the authors and do not necessarily represent those of their affiliated organizations, or those of the publisher, the editors and the reviewers. Any product that may be evaluated in this article, or claim that may be made by its manufacturer, is not guaranteed or endorsed by the publisher.
